# Facile extraction and characterization of calcium hydroxide from paper mill waste sludge of Bangladesh

**DOI:** 10.1098/rsos.220681

**Published:** 2022-08-10

**Authors:** Mohammad Robel Molla, Most. Hosney Ara Begum, Syed Farid Uddin Farhad, A. S. M. Asadur Rahman, Nazmul Islam Tanvir, Muhammad Shahriar Bashar, Riyadh Hossen Bhuiyan, Md. Sha Alam, Mohammad Sajjad Hossain, Mir Tamzid Rahman

**Affiliations:** ^1^ Industrial Physics Division, BCSIR Laboratories, Dhaka, Bangladesh; ^2^ Institute of Fuel Research and Development, Bangladesh Council of Scientific and Industrial Research, Dhaka, 1205, Bangladesh; ^3^ Fiber and Polymer Research Division, Bangladesh Council of Scientific and Industrial Research, Dhaka, 1205, Bangladesh; ^4^ Department of Chemistry, Comilla University, Kotbari, Cumilla 3506, Bangladesh; ^5^ Institute of Mining, Mineralogy and Metallurgy, Bangladesh Council of Scientific and Industrial Research, Joypurhat 5900, Bangladesh; ^6^ Department of Chemistry, Jahangirnagar University, Savar, Dhaka 1342, Bangladesh

**Keywords:** paper mill sludge (PMS), calcium hydroxide, FTIR, XRD, wavelength dispersive X-ray fluorescence, Raman spectroscopy

## Abstract

Herein, paper mill waste sludge (PMS) from two different sources has been investigated to extract calcium hydroxide, Ca(OH)_2_ by a facile and inexpensive extraction process. PMS samples, collected from local paper mill plants of Bangladesh, were the main precursors wherein HCl and NaOH were used for chemical treatment. The as-synthesized products were analysed by a variety of characterization tools including X-ray diffraction (XRD), Fourier transform infrared (FTIR) spectroscopy, Raman spectroscopy, scanning electron microscopy (SEM) and energy dispersive X-ray (EDX) elemental analyses. Our studies confirm that the extracted product contains Ca(OH)_2_ as a major content, albeit it also includes CaCO_3_ phase owing to the inescapable carbonation process from the surrounding environment. The particle size of the synthesized products is in the range of 450–500 nm estimated from SEM micrographs. The crystallite domain size of the same estimated from XRD analyses and was found to be approximately 47 and 31 nm respectively for product-A and product-B considering major (101) Bragg peak of Ca(OH)_2_. The yield percentage of the isolated products is about 65% for samples collected from both sources.

## 1. Introduction

Pulp and paper industries are generating a vast amount of pulp per year all around the world to fulfil the ever-growing demand for papers and packaging materials for diverse applications [1–4]. A suitable estimation reported that the production volume of paper and cardboard in developed countries was approximately 1000 metric tons in 2019 [5]. In 2017, the amount of global production of paper and cardboard reached approximately 419.7 million metric tons and was approximately 391.2–410.9 million metric tons from 2008 to 2016 [6]. During the production of paper and pulp processing, a huge amount of calcium carbonate (CaCO_3_) is used. In the process of paper and cardboard production, calcium carbonate is considered as most cost-effective material for manufacturing of high-quality paper and paperboard by substitution of other expensive minerals or wood additives. It is especially used to enhance the paper opacity and brightness. Consequently, a large volume of paper mill sludge (PMS) has been generated worldwide which contains an enormous amount of CaCO_3_. In many countries, except some well-renowned industries, a large number of pulp and paper industries are disposing this PMS without further utilizing/recycling/extracting calcium compounds, causing various types of ecological and environmental negative impacts. Therefore, it is a pressing necessity to utilize this PMS for value-added products as well as to address its negative impacts on our environment. On the other hand, calcium hydroxide (Ca(OH)_2_) materials have a variety of applications such as advanced bone repairing [7], de-acidification and wood conversion [8], protection of cultural heritage [9], calcium oxide (CaO) synthesis from Ca(OH)_2_ [10,11], use as a binding agent in the production of Portland cement [12,13], advanced application in the biomedical research [14], removal of phosphorus from aqueous medium [1], direct and indirect pulp processing [15], dental research [16] and so forth.

Numerous methods have been developed for the synthesis of calcium hydroxide such as precipitation [17], sol-gel-method [18], water-in-oil micro-emulsions [19], sono-chemical [20] and hydrogen plasma-metal reaction [21]. According to literature studied, Ca(OH)_2_ was synthesized by chemical precipitation (CP) process by different researchers [22–31] where salts of calcium chloride or nitrates and sodium hydroxide were the primary starting materials. Various types of waste materials were also used as calcium sources such as snail shell [32], eggshell [33], clamshell [34] and so on. Water-in-oil process [19], wet chemical process [35], heterogeneous phase synthesis [24] and moisture effect process [34] were also conducted by others. In the case of choosing synthesis medium, mainly three types of media were utilized such as aqueous [19,22,23,29,30,33], organic [19,24,25,27,28,31,35] and organic + aqueous [25,26]. Depending on reaction conditions, maintaining different temperatures above 90°C was noticed. From the literature cited above, it is also seen that synthesis of Ca(OH)_2_ by using CaCl_2_/Ca(NO_3_)_2_/waste materials and NaOH through various typical methods has been performed; however, utilizing waste PMS for synthesizing Ca(OH)_2_ has rarely been reported. Our objective of the present study is to extract Ca(OH)_2_ from PMS waste material and develop a facile and inexpensive process by chemical treatment or precipitation process at room temperature without utilizing any ionic/non-ionic surfactants in aqueous medium. Surfactant molecules have the propensity to stick on the surface of particles and the size as well as shape of particles may be affected by the concentration of surfactants and chemical nature [22,25,34]. Therefore, the main novelty of this study is the demonstration of a feasible method of Ca(OH)_2_ synthesis which has the potential for large-scale production. To that end, the experimental results are presented and discussed below.

## 2. Material and methods

### 2.1. Materials and reagents

Paper mill sludge (PMS) samples were collected from two different local paper mill plants of Bangladesh as calcium-containing source materials. Hydrochloric acid (37%) and sodium hydroxide (CAS: 1310-73-2, Purity approx. 98.0%) were purchased from Merck, Germany and DAEJUNG, Korea, respectively. Distilled water was used throughout the work as needed. All chemicals were utilized without further purification.

### 2.2. Synthesis methods

First of all, the collected PMS was mixed with distilled water to prepare a homogeneous mixture which was then filtered by a suction pump. After filtration, the mixture was dried in an electric oven at 60–65°C for 2 h in air until complete removal of water. Then it was crushed manually by using a ceramic mortar/pestle. Afterwards, a certain amount of dry-solid sludge was taken in a beaker, mixed with distilled water, and then stirred for 45 min. Meanwhile, 1.0 M HCl was added in solution to dissolve all the calcium contents in the aqueous medium, where pH of the sludge solution was maintained in the range of 2.25–2.50. After filtering the acidic solution, the filtrate part (very clear) was taken under base treatment by NaOH, maintaining a pH above 13.0 and the product formation/precipitation was seen to start within few minutes. The raw and synthesized samples were safely stored into the sample vials for various characterizations (a representative photograph of these samples is shown in electronic supplementary material, figure S1).

### 2.3. Characterization of materials

In order to find the characteristic functional groups in the as-synthesized products, the IR spectra of the samples were recorded in the range of 450–4000 cm^−1^. An FTIR spectrometer with a resolution of 4 cm^−1^ (Frontier, Perkin-Elmer, UK; Software v. 10.4.4.) and the typical potassium bromide (KBr) pellet technique was utilized for the same. The study of crystal structure along with mineral phase identification of samples were conducted by means of X-ray diffraction (EMMA GBC Corporation Company) using Cu *K_α_*_1_ (wavelength, *λ* = 1.54056 Å) source operated at 40 kV and 30 mA. The X-ray diffraction (XRD) data were recorded in the range of 2*θ* = 10°–80° with a step size of 0.05°. Raman spectroscopic measurements were performed at room temperature by a Horiba MacroRAM equipment using 785 nm diode laser (laser power less than 5 mW) as excitation source. A silicon wafer sample (Raman peak approx. at 520.7 cm^−1^) was used to calibrate thespectrometer prior to the data acquisition of the samples. The surface morphology and elemental composition of samples were conducted by a scanning electron microscope (SEM, Zeiss, EVO-18) coupled with an energy dispersive X-ray (EDX) spectrometer (AMETEK). Prior to synthesis of Ca(OH)_2_, the as-collected raw PMSs were characterized by a wavelength dispersive X-ray fluorescence (WD-XRF) equipment (Rigaku ZSX Primus) to investigate the inorganic contents in the samples.

## 3. Result and discussion

### 3.1. Synthesis

We have examined various batches of PMS samples for acid-base pH optimization and all the necessary information is listed in table 1. At the beginning of the treatment, the pH value in both acidic and basic media was not suitable and the amount of the yield was very low. When pH was kept between 2.25 and 2.50 (in acidic medium) and above 13.0 (in basic medium) [1,15,23,31], the amount of product was higher (table 2). In this work our optimized pH values during chemical treatment of sludge were 2.25–2.50 (in acidic medium) and above 13.0 (in basic medium). The Ca(OH)_2_ formation by the chemical precipitation route involves the following chemical reactions:

$$\mathrm{Sludge}\;(\mathrm{CaC}O_{3})+2{\mathrm{HCl}}_{\left(\mathrm{aqueous}\right)}\to {\mathrm{Ca}}_{\left(\mathrm{aqueous}\right)}^{2+}+2{\mathrm{Cl}}_{\left(\mathrm{aqueous}\right)}^{-}+{\mathrm{CO}}_{2\;(\mathrm{gas})}+H_{2}O_{\left(\mathrm{aqueous}\right)}$$

and

$${\mathrm{Ca}}_{\left(\mathrm{aqueous}\right)}^{2+}+2{\mathrm{Cl}}_{\left(\mathrm{aqueous}\right)}^{-}+2{\mathrm{NaOH}}_{\left(\mathrm{aqueous}\right)}\to \mathrm{Ca}{\left(\mathrm{OH}\right)}_{2\;(\mathrm{solid})}+2{\mathrm{Na}}_{\left(\mathrm{aqueous}\right)}^{+}+2{\mathrm{Cl}}_{\left(\mathrm{aqueous}\right)}^{-}$$

**Table 1.** Optimization of pH value to obtain the highest product during acid and base treatment of dried raw PMS. M, concentration of molar solution.

acid and base treatment of dry sludge for pH adjustment								
batch no.	weight of taken sample (g)	the volume of water added in the sample	HCl ml/M	pH (in HCl)	NaOH ml/M	pH (in NaOH)	weight of product, Ca(OH)_2_ (g)	weight of pulp residue (g)
1	2.0	300	205/0.5	2.87	234/0.5	12.18	0.1000	0.1700
2	2.0	250	55/1.0	2.52	99/1.0	12.64	0.2295	0.2701
3	2.0	250	40/1.0	2.28	200/1.0	13.03	1.0615	0.2614
4	2.0	200	42/1.0	2.10	100/2.0	13.09	1.0462	0.2500
5	2.0	200	50/1.0	1.90	125/2.0	13.05	1.0812	0.1872
6	2.0	150	45/1.0	2.11	220/2.0	13.13	1.1400	0.2620
7	2.0	125	46/1.0	2.00	155/3.0	13.42	1.1420	0.2690
8	2.0	125	45/1.0	2.01	200/3.0	13.44	1.2100	0.2730
9	2.0	125	45/1.0	2.00	350/3.0	13.56	1.1500	0.2610

**Table 2.** Wavelength dispersive X-ray fluorescence (WDXRF) characterization of PMS sample source-A and sample source-B.

components	source-A (wt%)	source-B (wt%)
Na_2_O	0.0955	0.0299
MgO	0.2808	0.2916
Al_2_O_3_	0.7067	0.4357
SiO_2_	1.1749	1.6297
P_2_O_3_	0.0224	0.0190
SO_3_	0.0783	0.0506
NaCl	0.0968	0.0592
K_2_O	0.0317	0.0395
CaO	95.9056	94.6093
Cr_2_O_3_	0.2999	0.6586
MnO	—	0.1226
Fe_2_O_3_	1.1674	1.9348
ZnO	0.0201	0.0388
Rb_2_O	—	0.0083
SrO	0.0281	0.0355
ZrO_2_	0.0740	0.0249
Nb_2_O	—	0.0120
TiO_2_	0.0178	—
Total	100.0000	100.0000

In the first batch, the molar concentration of HCl and NaOH was 0.5 M which was considered for examining the effects of different concentrations (HCl and NaOH) on the amount of product formation (table 1). Finally, 1.0 M concentration of HCl and 3.0 M concentration of NaOH were chosen. The variation of volume of water shown in table 1 was only to minimize the amount of water used during sample preparation. Sodium hydroxide (NaOH) was used as a precipitator. During insertion of NaOH solution, continuous stirring at a rate of 1300 r.p.m. at room temperature was maintained. After complete precipitation, the product was filtered by a Whatman 40 (GE Healthcare UK Limited, Little Chalfont, Buckinghamshire, UK) paper. For removal of NaCl, the product was washed several times with deionized water, after which it was dried in an electric oven at 65°C–70°C for 3 h and preserved in airtight sample bottles. The residual part obtained from acid solution filtration was collected, dried and kept for further research work (e.g. activated carbon).

At optimized pH value (2.25–2.50, in acid and above 13.0, in base) five batches of acid and base treatment for calcium hydroxide isolation were performed for sample (a) and sample (b) (electronic supplementary material, table S1). The batch numbers were denoted as S-a A, S-a B, S-a C, S-a D and S-a E for sample (a) and S-b A, S-b B, S-b C, S-b D and S-b E for sample (b). For each sample, a different amount of raw sludge was taken (2.0, 4.0, 6.0, 8.0 and 10.0 g) for batches A, B, C, D and E, respectively. The amount of water was not fixed and our attempt was to minimize or reduce usage of water. For sample (a) of the first batch, the amount of product was 0.07 g, which was found to be greater than that of sample (b). However, for other batches the amount of product for sample (b) was higher than sample (a) (see electronic supplementary material, table S1).

**Figure 1.**
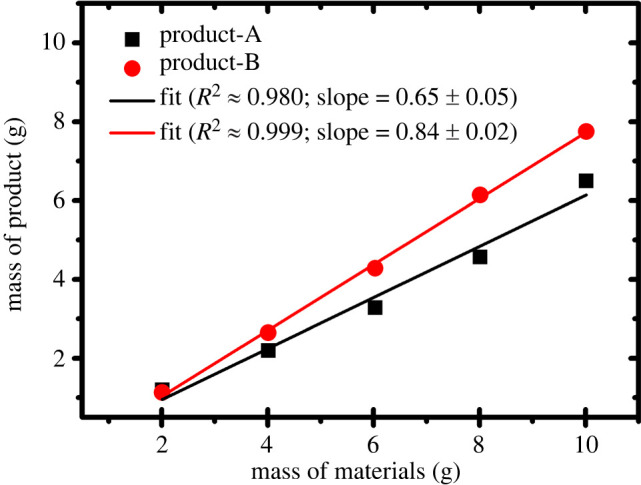
A correlation plot of the mass of raw materials taken versus the mass of isolated product-A and product-B from different sampling batches.

With the increased amount of raw PMS, the amount of product materials was found to be gradually increased (see electronic supplementary material, table S1), which is illustrated in figure 1.

According to figure 1, it can be anticipated that the higher amount of starting sludge will provide the expected amount of calcium, and this process is expected to be viable in the industrial production. The average percentage of yield is about 65%, out of 2 g sample. The product obtained from sample (a) and sample (b) is denoted as the product-A and product-B respectively. From figure 1, it can also be inferred that Ca-compounds yield in product-B (slope = 0.84 ± 0.02) is higher compared with product-A (slope = 0.65 ± 0.05). However, the standard deviation (s.d.) of mass production per unit raw materials usage in each batch is approximately 0.04 for product-A and approximately 0.09 for product-B.

Owing to using waste material for the extraction of Ca(OH)_2_, it is very important to pre-investigate the raw sludge for identifying its chemical constituents; therefore, mineralogical studies by means of X-ray fluorescence were also performed. The obtained results are in oxide form and illustrated in table 2 where CaO is in the highest amount: 95.9056 (wt%) for source-A and 94.6093 (wt%) for source-B of raw PMS materials.

Apart from the major component (CaO), other components were also found in trace level, except SiO_2_ and Fe_2_O_3_ which in combination were approximately 2.4%. This huge amount of calcium content in PMS motivated us to find a facile extraction process of calcium hydroxide which was discussed in the Material and methods section above. The extracted products were then systematically characterized by various characteristic tools and discussed below.

### 3.2. Surface morphology and chemical composition of the isolated product

**Figure 2.**
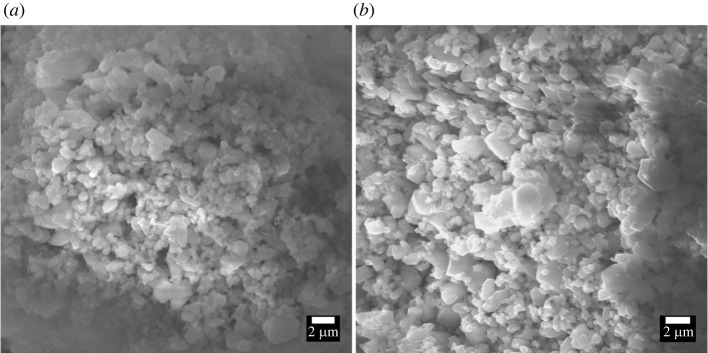
Surface morphology of isolated product-A (*a*) and product-B (*b*).

The morphological features of the obtained product-A and product-B were explored by means of scanning electron microscopy (SEM), and their SEM micrographs are shown in figure 2.

**Figure 3.**
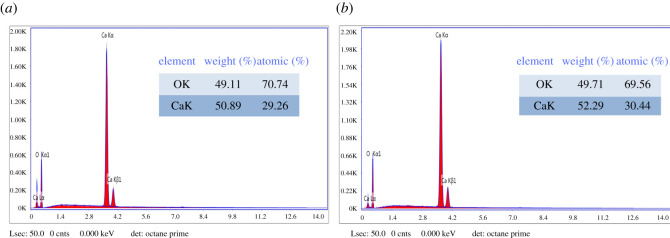
EDX microanalysis of isolated product-A (*a*) and product-B (*b*). Their elemental compositions are shown in the inset tables.

The SEM micrographs exhibit that the powder grains/particles in the synthesized products are polygons but with no uniform shape. The average particle size was determined by ImageJ software and it was found to be in the range of 450–500 nm for both products. Numerous studies in the literature revealed the formation of nano-calcium hydroxide with approximately similar morphologies and size [27,28]. The elemental composition of product-A and product-B was also investigated by SEM/EDX microanalyser and is shown in figure 3.

In product-A and product-B, calcium content is 50.89% and 52.29% respectively and oxygen content is 49.11% and 47.71% respectively. These values (weight %) are summarized in two tables inside the respective figure (cf. figure 3). From the EDX microanalyses, it is evident that calcium-based compounds in product-B are slightly greater than that of the product-A corroborating the results shown in figure 1 above.

### 3.3. FTIR analyses

**Figure 4.**
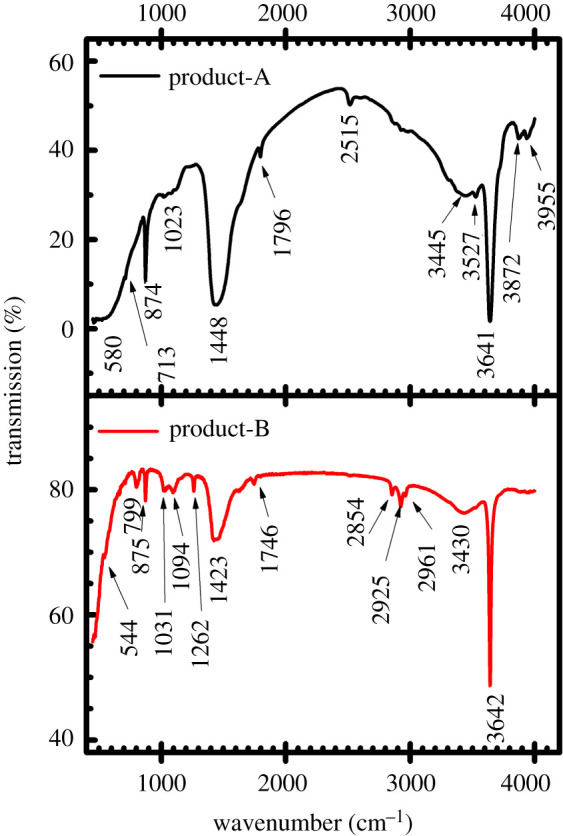
FTIR spectra of the isolated product-A and product-B from two different PMS sources.

Figure 4 depicts FTIR patterns of the obtained product-A and product-B, where both spectra are approximately similar but with a little difference regarding the peak intensities. The reference FTIR curves of calcium-based compounds from RRUFF database [36] can be found in electronic supplementary material, figure S2.

The relatively strong absorption band approximately at 3641 cm^−1^ (product-A) and 3642 cm^−1^ (product-B) corresponds to the stretching mode of hydroxyl group (OH) [1,23,37] (also see electronic supplementary material, figure S2). In addition, there is a possibility of some water molecules incorporation on the sample surface from the air during sample handling [38]. The (OH) stretching band is noticeably sharp and may signify the pure calcium hydroxide phase [23]. The broadband peaks approximately ranging from 3430 to 3527 cm^−1^ also reveal the existence of corresponding OH stretching modes. Some common peaks, clustered approximately from 2515 to 2961 cm^−1^ and from 1746 to 1796 cm^−1^, have been attributed to the adsorption of atmospheric CO_2_ and stretching mode of C=O bond, respectively [23]. The broad stretching absorption and sharp peaks approximately at 713 and 799 cm^−1^, 874 and 875 cm^−1^, and 1448 and 1423 cm^−1^ (cf. product-A and product-B) respectively represents ʋ_4_ (in-plane-bending mode/bending vibration), ʋ_2_ (out-of-plane bending mode/symmetric deformation), and ʋ_3_ (antisymmetric stretching mode) of carbonate group (${\mathrm{CO}}_{3}{\;}^{2-}$) of the calcite [1,33,39].The peak value ranging approximately from 1023 to 1094 cm^−1^ is because of ʋ_1_ (symmetric stretching mode) for the CO_3_^2−^ group in calcite [23,31]. In figure 4 (product-B), peak value approximately at 1262 cm^−1^ corresponds to the stretching mode of C–O bond in the ${\mathrm{CO}}_{3}{\;}^{2-}$group [40]. The wide and strong band peaks approximately at 580 and 544 cm^−1^ in figure 2 illustrate the presence of Ca–O band of symmetric vibration [33,39]. Additionally, the vibrational peaks approximately at 3872 and 3955 cm^−1^ in product-A indicate the vibrational mode of O–H [41]. In summary, FTIR analyses suggest that both Ca(OH)_2_ and CaCO_3_ are present in the synthesized products [23,24].

### 3.4. XRD analyses of PMS source and products

**Figure 5.**
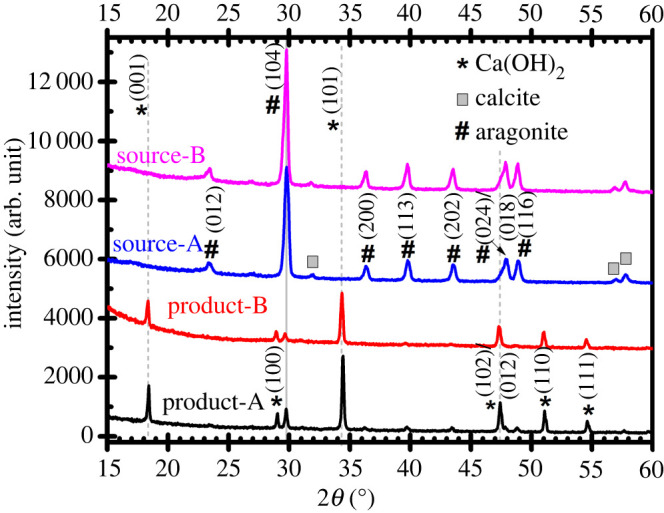
(vertically offset for clarity) XRD patterns of isolated product-A, product-B, source-A and source-B. Aragonite (#) and calcite (□) are the two polymorphs of CaCO_3_.

The XRD patterns of representative PMS source and synthesized products are illustrated in figure 5 and the (hkl) reflection peaks are matched with the diffraction peaks of the portlandite (hexagonal Ca(OH)_2_, marked by *); calcite (rhombohedral CaCO_3_, marked by □); and aragonite (orthorhombic CaCO_3_, marked by #) collected from the RRUFF database [36]. The XRD patterns of these calcium-based compounds can be found in electronic supplementary material, figure S3.

From figure 5, it is clear that PMS samples collected from source-A and source-B were mainly composed of aragonite phase of CaCO_3_ with small amount of calcite phase of CaCO_3_ evident from the minor Bragg peaks (marked by □) in the range of 2*θ* = 30°–60°. No XRD peaks related to Ca(OH)_2_ are detected. (Full scanning range (2*θ* = 10°–80°) XRD patterns of all samples can be found in electronic supplementary material, figure S4.) By contrast, XRD patterns of both product-A and product-B exhibit diffraction peaks (marked by *) corresponding to the hexagonal phase of Ca(OH)_2_ having space group P-3m1 (Space Group no. 164, PDF Card No. 00-087-0673) [1,23,31]. For product A/B, these diffraction peaks appeared at 2*θ* = 18.42°/18.34°, 29.04°/28.94°, 34.44°/34.36°, 47.44°/47.32°, 51.10°/51.04°, 54.62°/54.52°, 62.88°/62.76°, 64.52°/64.42° and 72.00°/71.88° corresponded to the (001), (100), (101), (102), (110), (111), (021), (013) and (002) planes of the Ca(OH)_2_. In addition, a minor Bragg peak appeared approximately at 2*θ* = 29.8° which can be attributed to the #(104) plane of the orthorhombic CaCO_3_ phase (denoted by solid line). Numerous studies in the literature ([1,23,24,31] and references therein) reported that the inevitable generic presence of Ca(OH)_2_ and CaCO_3_ phase is due to the reaction of atmospheric CO_2_ with Ca(OH)_2_ (*aka* carbonation process) irrespective of the synthesis routes. However, in our study, taking the ratio of area under curves [42] of peak *(101) and #(104) suggest that Ca(OH)_2_ content in product-B is 1.84 times higher compared with that of the product-A (see electronic supplementary material, table S2). These observations again suggest that synthesized products from PMS source are mainly composed of Ca(OH)_2_ phase with small amount of CaCO_3_ phase. This is consistent with the results presented in figures 1 and 4.

In order to elucidate the mean crystallite domain size (*d)* of the synthesized product, the Scherrer's equation (3.1) [43] was utilized.

**Figure 6.**
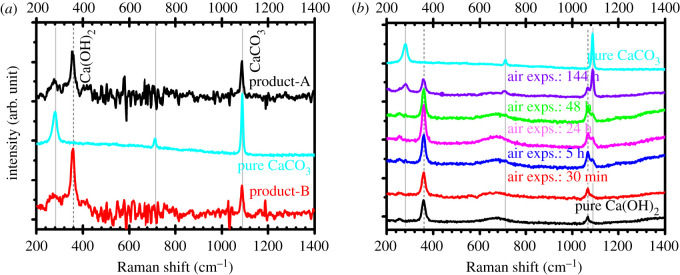
Room temperature Raman spectra of (*a*) the isolated product-A, product-B, and a reference CaCO_3_ (purity approx. 99.95%) sample; (*b*) pure Ca(OH)_2_ (purity approx. 99.95%) and the same Ca(OH)_2_ with various air exposure duration (30 min, 5 h, 24 h, 48 h and 144 h). The solid and dashed vertical lines indicate the reference Raman peaks of CaCO_3_ and Ca(OH)_2_ respectively.

3.1 $$d\;=\frac{K\lambda }{\beta \cos\theta },$$

where *K* is Scherrer's constant, equal to 0.94, *λ* is the wavelength of X-ray radiation used (*λ* = 1.5406 Å), *θ* is the Bragg diffraction angle and *β* is the full width at half maximum (FWHM) in radiation. The most prominent *(101) peak of Ca(OH)_2_ was considered to estimate the mean crystal domain sizes and was found to be 41.96 nm (for product-A) and 36.49 nm (for product-B). While considering #(104) peak of CaCO_3_, mean crystallite domain size was found to be 39.20 nm (for product-A) and 32.15 nm (for product-B) (cf. electronic supplementary material, figure S5 and table S2). It can be seen that diffraction peaks for product-A are slightly shifted to higher 2*θ* values compared with that of product-B (indicated by the major diffraction peaks of Ca(OH)_2_ denoted by dashed line in figure 6), which is presumably due to the stress–strain effect owing to the combined presence of Ca(OH)_2_ and CaCO_3_. The lattice strain, *ε* of crystal at the plane *(101) and #(104) were determined using the following expression (3.2) [43]:

3.2 $$\varepsilon \;=\frac{\beta }{4\tan\theta }.$$

The calculated value of the lattice strain was found to be 2.92 × 10^−3^ (product-A) and 3.36 × 10^−3^ (product-B) considering the plane *(101) of Ca(OH)_2_ and 2.60 × 10^−3^ (product-A) and 4.40 × 10^−3^ (product-B) while considering the plane #(104) of CaCO_3_. In both cases, microstrain in product-B is higher than that of product-A. To elucidate the origin of minor presence of CaCO_3_ phase in the Ca(OH)_2_ compound and therefore, their stress/strain-related effect, we have performed the Raman spectroscopic analyses systematically, as discussed in the §3.5 below.

### 3.5. Raman spectroscopic analysis

Raman spectra of the isolated product-A, product-B, pure CaCO_3_ and pure Ca(OH)_2_ samples were recorded at room temperature and maintaining the same experimental conditions (i.e. same laser exposure time: 5 s, no. of accumulations: 5, laser power less than 5 mW, spot size diameter is approximately less than 0.5 mm) and these spectra are shown in figure 6. The Ca(OH)_2_ samples were left in open laboratory air for various time of durations and then Raman spectra were recorded after 30 min, 5 h, 24 h (1 day), 48 h (2 day), 144 h (6 day) duration for monitoring the CaCO_3_ formation kinetics within the Ca(OH)_2_ materials (figure 6*b*).

The Raman spectrum of the pure CaCO_3_ samples exhibited vibrational peaks (solid lines) approximately at 157.18, 280.95, 713.46 and 1087.53 cm^−1^ and pure Ca(OH)_2_ samples exhibited vibrational peaks (dashed lines) approximately at 359.72 cm^−1^ (357.66 cm^−1^ for 144 h air exposed Ca(OH)_2_ sample) and 1066.78 cm^−1^, which are very much consistent with their respective Raman spectrum from RUFF database (cf. figure 6 and electronic supplementary material, figure S2b). As can be seen from figure 6*a*, the Raman peaks at 353.53 and 1085.65 cm^−1^ are seen for product-A, while at 357.66 and 1085.65 cm^−1^ are seen for product-B. These peak positions can be attributed to the Raman peak value of Ca(OH)_2_ (approx. 360 cm^−1^) and CaCO_3_ (approx. 1088 cm^−1^) respectively. Notice also that peak size of Ca(OH)_2_ in product-B is larger than that of product-A, suggesting a higher amount of calcium hydroxide in product-B compared with that of product-A, corroborating the results shown in figures 1, 3 and 5. Additionally, the Ca(OH)_2_ peak in product-A is approximately 4 cm^−1^ red-shifted compared with that of product-B. This indicates the higher microstrain induced in product-B compared with product-A corroborating the XRD results (see electronic supplementary material, figure S6). This is presumably due to the higher amount of CaCO_3_ inclusion in product-B compared with product-A. Referring to the XRD analyses, we observed that Ca(OH)_2_ Bragg peaks in both groups of products is significantly higher than that of CaCO_3_ (figure 5)_._ Numerous studies reported that the inclusion of CaCO_3_ in air-exposed Ca(OH)_2_ is inevitable due to the interaction of atmospheric CO_2_ with Ca(OH)_2_ [44,45]. In figure 6*b*, we verified this inevitable carbonation process by analysing Raman spectra of a pure Ca(OH)_2_ sample which was systematically exposed in air for various durations. Notice that with the increase of air-exposure durations, the Ca(OH)_2_ peak approximately at 1066.78 cm^−1^ (dashed line) is consistently decreasing with the increasing of the CaCO_3_ peak (solid line) appeared approximately at 1087.53 cm^−1^. With increasing CaCO_3_ peak in the Ca(OH)_2_ sample, major Raman peak of Ca(OH)_2_ at approximately 360 cm^−1^ is slightly red-shifted compared with the pure sample (see electronic supplementary material, figure S3 for details). In summary, from all experimental results shown above, we can confirm that our extraction process yielded Ca(OH)_2_ phase as major product from the two different PMS sources, and the minor inclusion of CaCO_3_ phase in the products is due to their air-exposure in the laboratory, which could be avoided by performing the extraction process either in the air-tight chamber or in the inert atmosphere. However, it can be inferred from the FTIR, XRD and Raman analyses that the product-B is comparatively purer than the product-A in terms of Ca(OH)_2_ content.

## 4. Conclusion

Our study demonstrates a facile extraction process of calcium hydroxide materials from paper mills sludge collected from two different sources. To the best of our knowledge, we have utilized paper mill's waste of Bangladesh for the first time to extract this valuable chemical. In addition, our extraction process was accomplished in water at room temperature by a common, low energy-intensive and cost-effective chemical precipitation method without using any ionic and non-ionic surfactants. Due to the well-known carbonation process, a small presence of CaCO_3_ phase was detected by FTIR, Raman and XRD. However, all the presented results conducted by a variety of characterizations tools conspicuously reveal that synthesized products from the both PMS sources are mainly composed of calcium hydroxide. Only acid and base treatment with a certain range of pH in the two different media gives our method a great potential to implement in the recycling sectors of pulp and paper industries for the extraction of valuable calcium-based compounds as well as recycling the waste sludge.

## Data Availability

The datasets which are supporting this article have been uploaded as the electronic supplementary material [46].
